# Nutritional Status and Its Association with Possible Sarcopenia in Older Homeless Adults

**DOI:** 10.1093/geroni/igaf122.2435

**Published:** 2025-12-31

**Authors:** Phatcharaphon Whaikid, Noppawan Piaseu

**Affiliations:** Mahidol University, Bangkok, Krung Thep, Thailand; Ramathibodi Hospital, Bangkok, Krung Thep, Thailand

## Abstract

This study assesses nutritional status and possible sarcopenia among formerly homeless adults aged 50 years and older residing in Thai supportive housing. A total of 284 older homeless adults were screened, of whom 116 met the inclusion criteria and were selected using purposive sampling. Data were collected on sociodemographic characteristics, nutritional status (assessed using the Mini Nutritional Assessment-Short Form [MNA-SF]), and possible sarcopenia (determined using the AWGS 2019 criteria), including calf circumference (< 34 cm in men, < 33 cm in women), low handgrip strength (< 28 kg in men, < 18 kg in women), or low gait speed (≤1.0 m/s). Data were analyzed using descriptive statistics and logistic regression analysis. Among the participants, 66.4% were male, with a mean age of 59.14 years (SD = 7.79). Overall, 78.4% of participants were classified as having possible sarcopenia. Univariate analysis showed that malnutrition (OR = 6.111, 95% CI = 2.104–17.750), body mass index (BMI) (OR = 0.031, 95% CI = 0.009–0.100), and waist circumference (WC) (OR = 0.129, 95% CI = 0.049–0.342) were significant predictors of possible sarcopenia. Multiple logistic regression analysis indicated that malnutrition (OR = 3.860, 95% CI = 1.068–13.946) and BMI (OR = 0.038, 95% CI = 0.011–0.128) together accounted for 53.3% of the variance in predicting possible sarcopenia. Findings highlight a high prevalence of possible sarcopenia, with malnutrition and BMI as key risk factors. Targeted nutritional and muscle health interventions are essential to mitigate sarcopenia risk in this vulnerable population.

